# Examination of Myofascial Stiffness and Elasticity in the Upper Trapezius Region in Patients with Unilateral Neck Pain: A Cross-Sectional Study

**DOI:** 10.3390/jcm12196339

**Published:** 2023-10-03

**Authors:** Anika Seidel, Andreas Brandl, Christoph Egner, Robert Schleip

**Affiliations:** 1 Department of Medical Professions, Diploma Hochschule, 37242 Bad Sooden-Allendorf, Germany; 2 Department of Sports Medicine, Institute for Human Movement Science, Faculty for Psychology and Human Movement Science, University of Hamburg, 20148 Hamburg, Germany; 3 Conservative and Rehabilitative Orthopedics, Department of Sport and Health Sciences, Technical University of Munich, 80333 Munich, Germany

**Keywords:** unilateral neck pain, myofascial stiffness, indentometry, pain pressure threshold

## Abstract

(1) Background: Globally, neck pain is prevalent, affecting around thirty percent of the population annually. To better understand the influence of pain on the myofascial layers, the present study investigated these on the upper trapezius muscle in unilateral, more severe neck pain. (2) Methods: This study was a cross-sectional study. Forty patients (42.2 ± 14.7) with a confirmed diagnosis of unilateral neck pain were examined using durometry and indentometry. This study evaluated the stiffness, elasticity, and pressure pain threshold of both sides of the neck (symptomatic side: SS; healthy side: HS). Furthermore, the range of motion of the cervical spine (lateral flexion, rotation) was quantified using a digital goniometer. (3) Results: A significant lateral discrepancy was observed in stiffness between groups (durometry: SS—33.76 ± 7.78, HS—29.75 ± 7.45, *p* < 0.001; indentometry: SS—59.73 ± 33.93, HS—4.18 ± 12.69, *p* = 0.024). In contrast, no differences were found between the comparison sides of the upper trapezius for the parameter’s elasticity (SS—0.101 ± 1.09, HS—−0.006 ± 0.29, *p* = 0.416), cervical spine mobility (lateral flexion: SS—37.08 ± 8.15, HS—37.73 ± 7.61, *p* = 0.559; rotation: SS—73.55 ± 12.37, HS—72.85 ± 11.10, *p* = 0.660), and algometry (SS—36.41 ± 17.53, HS—37.22 ± 17.00, *p* = 0.657). (4) Conclusion: Overall, it can be concluded that more severe neck pain unilaterally shows differences in stiffness on the same side. Future research is needed to investigate the links.

## 1. Introduction

According to the Task Force for Neck Pain, neck pain affects an estimated 12.1 to 71.5 percent of the population every year. For the working population, this figure is in the range of 27.1 to 47.8 percent. As neck pain follows a lifelong sinus curve with alternating epi- and remissions, it is estimated that between 50 to 85 percent of those affected will experience a new pain episode within the next one to five years [1].

Previous studies have focused on general neck pain or the effect of pain on other regions, such as the lower trapezius. For example, it is already known that tension and overactivity of the upper trapezius, which tends to shorten and form trigger points, leads to weakness in the middle and lower parts [2]. In one of his publications, Choudhari et al. [3] found that in unilateral neck pain, the strength of the middle and lower trapezius is significantly reduced compared to the contralateral side. A second study assessed the strength capacity of the trapezius muscle in violinists with unilateral neck pain and showed a deficit in the parameter studied on the painful side of the neck [4]. Looking at the thickness of the lower trapezius in patients with unilateral neck pain, it is reduced both at rest and during contraction compared to the control group [5].

There have also been studies contrasting healthy individuals with those who suffer from neck pain. In terms of stiffness, for example, it has been shown that people with chronic neck pain have a significantly stiffer trapezius muscle than healthy people [6,7]. There also appears to be a linear, positive correlation between penetration force and elastomer stiffness for this parameter [8]. Kocur et al. [9] also describe a positive correlation between stiffness, age, body mass index (BMI), and trapezius elasticity.

However, there are no known trials that compare side-dependent measurement values of neck pain in the same person. This ensures better comparability and helps to determine how unilateral, severe neck pain affects the myofascial tissue properties of the upper trapezius in comparison to the opposite side. The importance of researching the myofascial tissue properties related to neck pain is evident as it can reduce work absenteeism, lower healthcare expenses, and alleviate pressure on national healthcare systems.

The purpose was to investigate whether biomechanical tissue properties, pressure painfulness, as well as active mobility on the affected side of the neck show detectable differences between the right and left upper trapezius.

## 2. Materials and Methods

### 2.1. Design and Ethics

This study was a cross-sectional study with one group according to the STROBE guidelines [10]. The protocol was registered in the German Register for Clinical Studies (DRKS-ID: DRKS00031247). The measuring devices and the treatment instrument were granted by the Fascia Research Project of the Technical University of Munich and Ulm University in Germany. This study (No.: EB 1037/2022) obtained permission from the DIPLOMA Ethics Committee. All research procedures complied with the ethical principles of the Declaration of Helsinki. Each participant provided written informed consent at the outset of the study.

### 2.2. Participants

Patients between the ages of 18 and 75 with a confirmed clinical diagnosis of neck pain according to the ICD classification on one side of the body more than the other, as measured by a minimum difference of three on the Visual Analogue Scale (VAS), were included. The neck pain should be acute, i.e., not more than six months old. Exclusion criteria were previous surgery or other scars in the neck region between C1 und Th1, use of medication that affects the muscle tone (e.g., analgesics and muscle relaxants), rheumatic diseases, skin changes (e.g., neurodermatitis, psoriasis) in the upper trapezius area, and/or pregnancy. Based on these criteria, *n* = 40 subjects from a middle-size city in middle Germany were recruited for the measurements.

### 2.3. Measurements

**Figure 1:**
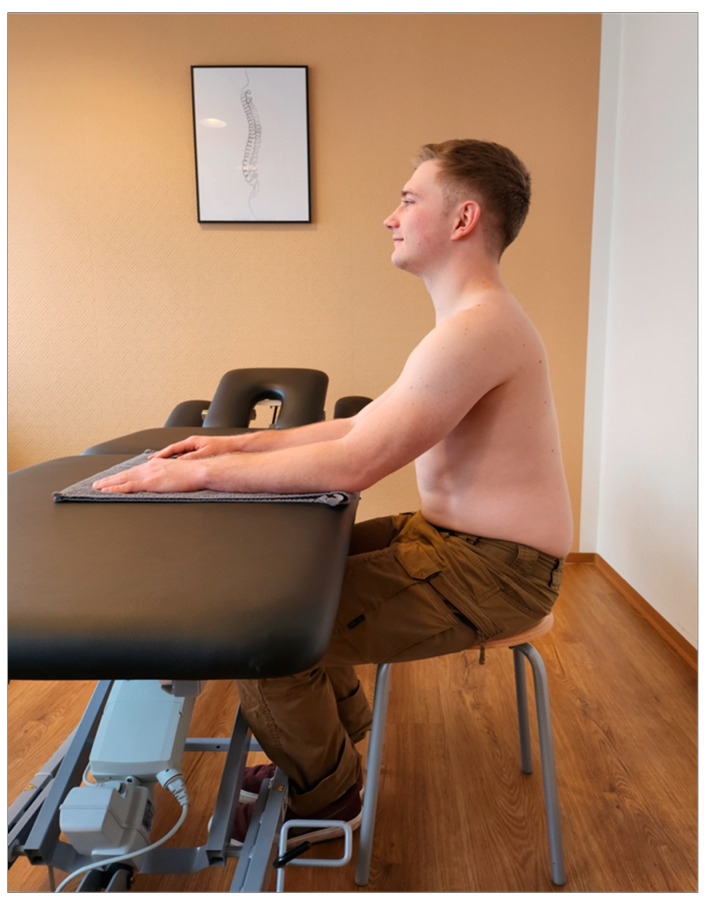
Initial position of the test series.

Setting: Before starting the upper trapezius measurements, patients were asked to complete a short data sheet (age, sex, height, weight, pain intensity on the affected side using VAS). In addition, the correlation between neck pain and headache was assessed using a subtest of the Neck Disability Index assessment tool [11]. All examinations were performed by the same person and took approximately fifteen minutes. Sitting on a stool with forearms parallel and shoulder-width apart on a height-adjustable therapy couch (or table) was found to be an appropriate starting position. The height of the table was adjusted so that the top was approximately at the level of the patient’s thoracolumbar junction, and patients were asked to place only the weight of their arms, not their upper body, on the table. A towel was used to guide the patient. The upper body was aligned perpendicular to the floor, with the eyes forward and the shoulders not raised. The feet should be parallel and in firm contact with the floor (Figure 1).

**Figure 2:**
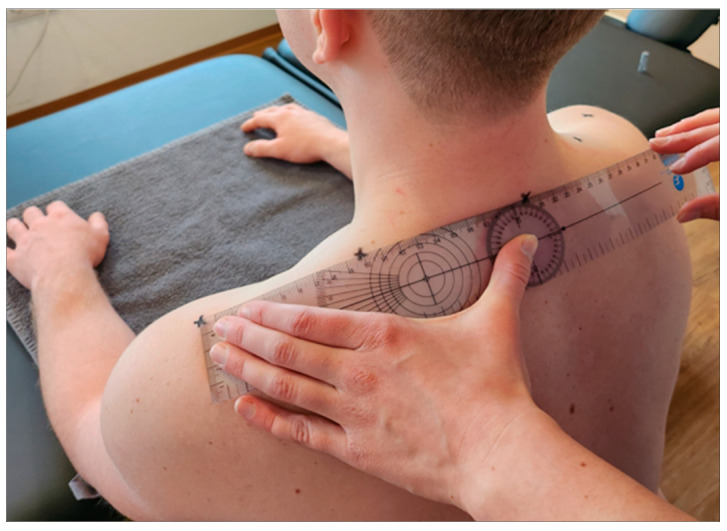
Determining the measurement point.

The measurement point was marked by the investigator and represented the midpoint between the processus spinosus of the 7th cervical vertebra and the tip of the acromion [12,13] (Figure 2). All subsequent measurements were taken on alternating sides to allow the tissue to recover for one minute at a time.

Durometer: Various definitions of the parameters listed above can be found in the literature. In this context, the term ‘tone’ is often used. However, this term is not easy to define physiologically. Nonetheless, it comprises two vital properties: stiffness and elasticity. Stiffness describes how the muscle recovers its original shape after manual compression with the former property. Resistance remains independent of compression in rapidly repeated cycles [14,15]. The subsequent examination series measure these properties by applying vertical pressure above the skin and observing the resistance or deformation of the muscle [12].

**Figure 3:**
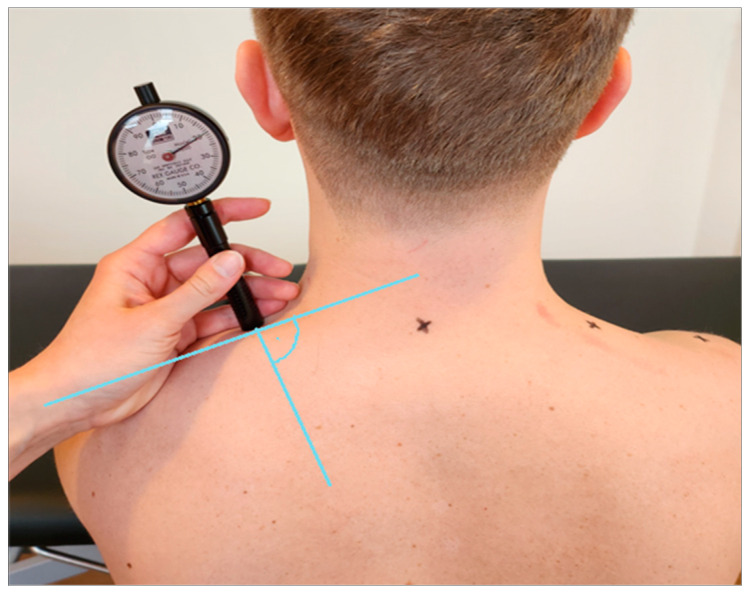
Measurement of stiffness and elasticity by durometer.

The first measurement was taken using a Shore durometer (Rex Gauge, Canada, ICC 6 mm = 0.77) [16], which serves as a reference device and measures surface hardness, i.e., the depth of penetration into a material, based on the force generated on a standardised measuring attachment [17]. Five consecutive measurements were taken on both sides at five-second intervals. The plunger sank into the myofascial tissue due to the inherent gravity of the device (Figure 3).

**Figure 4:**
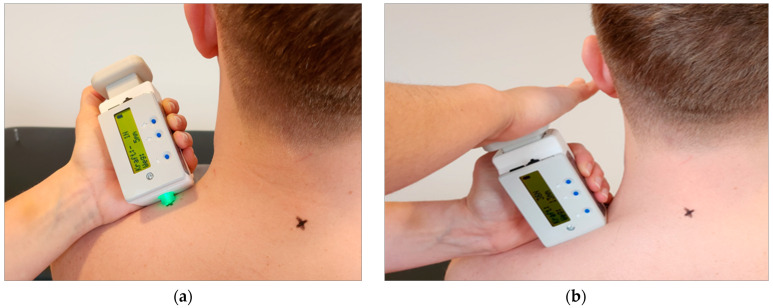
IndentoPro. (**a**) Attaching the IndentoPro measuring stamp; (**b**) measurement of stiffness and elasticity by IndentoPro.

*IndentoPro:* Fascial tissue properties (stiffness, elastic storage capacity, pressure pain threshold) were then determined using the IndentoPro (Fascia Research Group, Germany, ICC 6mm = 0.98) [16] (Figure 4). Myofascial elasticity refers to the capability of maintaining the same tissue stiffness following mechanical deformation repetition. Thus, it is the opposite of high viscoelastic relaxation, i.e., stiffness decreases over time, or high damping capacity [14,18,19]. The IndentoPro records the tissue’s elastic storage capacity when subjected to repeated compression. Hence, taking multiple measurements within a span of thirty seconds can evaluate the tissue under examination’s elastic storage capacity.

The predetermined penetration depth was five mm [20]. For stiffness, the measuring punch was pressed three times in thirty seconds and for elasticity, five times in ten seconds (10 N/s) into the previously marked points until the predefined indentation depth was reached [21]. The difference between the first and fifth measurement was used to determine the compressive elasticity of the tested tissue; the smaller the difference, the more elastic the tissue can be assumed to be.

To quantify algometry, the investigator used the pressure pain threshold (PPT). This measurement parameter is determined by applying the weakest pressure stimulus perceived as painful to the upper trapezius muscle [22,23]. This involved pressing the IndentoPro plunger into the myofascial tissue until the patient reported an additional pain sensation in addition to the mechanical pressure sensation. At this point, the participant was asked to say the word ’now’. The QST guidelines were used as a guide for the related measurement protocol [24].

**Figure 5:**
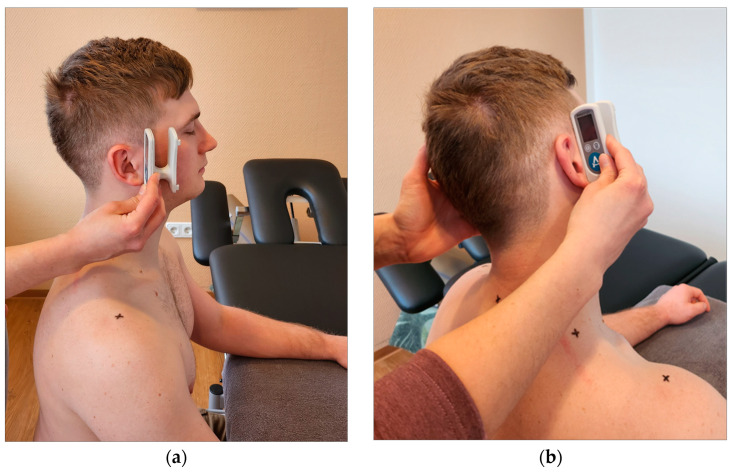
Lateral flexion. (**a**) Handling of the EasyAngle during lateral flexion of the cervical spine; (**b**) measurement of lateral flexion using EasyAngle.

**Figure 6:**
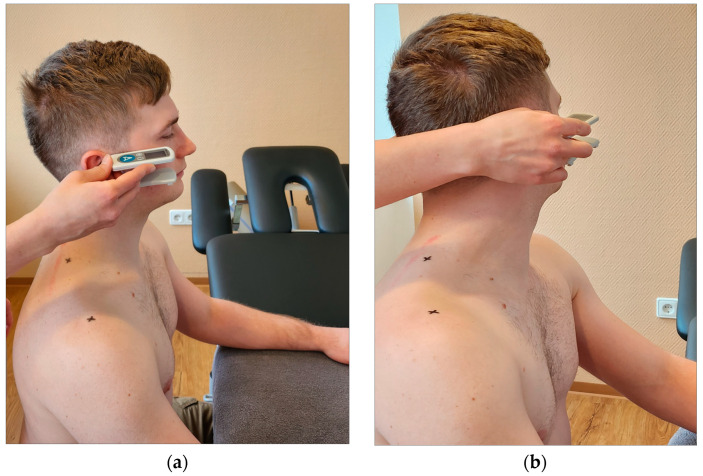
Rotation. (**a**) Handling of the EasyAngle during rotation of the cervical spine; (**b**) measurement of rotation using EasyAngle.

*Digital goniometer:* Finally, cervical spine mobility was quantified using a digital goniometer (Meloq AB, Sweden, ICC = 0.66) [25]. It was placed ventral to the subject’s ears and the subject was asked to actively tilt the head sideways toward the shoulder (Figure 5) or rotate the head (Figure 6). Any evasive movement during the ventral lateral tilt or torso rotation was corrected and prevented by the experimenter [25].

All measurements were painless. If the participant felt uncomfortable at any time during the examination series, he or she could indicate this by giving a hand signal, and the examination was then stopped.

### 2.4. Data Analysis and Statistics

The standard deviation, mean, and 95% confidence interval were determined to evaluate the measurement results. In addition, the Pearson product-moment correlation was recorded for the following parameters: durometer and IndentoPro, as well as age groups and lateral flexion, age groups and rotation, and age groups and elasticity; each for the symptomatic and non-symptomatic side. Somers’ D was used for correlations between the following ordinal parameters: pain (VAS) and headache frequency, pain (VAS) and headache intensity, gender and headache frequency, gender and headache intensity, and handedness and pain side. For multiple comparisons, the *p* value was adjusted using the Bonferroni correction. Correlations were interpreted according to Cohen as ‘weak’ (>0.09, <0.30), ‘moderate’ (>0.29, <0.50), and ‘strong’ (≥0.50) [26]. Comparisons between the symptomatic and non-symptomatic side were evaluated using a paired Student’s *t*-test. There were no outliers, i.e., more than three times the interquartile range of the upper or lower quartile, in the dataset. Normal distribution was assumed according to the central limit theorem [27]. Data were normally distributed according to the Shapiro–Wilk test (*p* > 0.05). The significance level was set at *p* = 0.05. Descriptive statistics were performed using Libreoffice Calc version 6.4.7.2, Mozilla Public License v2.0. Inferential statistics were performed using R software, version 3.4.1., R Foundation for Statistical Computing, Vienna, Austria.

## 3. Results

### 3.1. Study Group Characteristics

Of the 48 participants in the study, 40 met the inclusion criteria. All epidemiological data are presented in Table 1.

### 3.2. General Correlations between Age, Gender, Pain, and Mobility

Table 2 shows the extent to which there are general correlations between pain and the comparison variables. Lateral flexion and rotation were more clearly correlated on the more painful side, i.e., the older the participants, the more restricted their cervical spine mobility was. The comparison between age and elasticity on the more painful side of the neck was also significant (*p* = 0.048). The older the subjects were, the less elastic the myofascial tissue was on the painful side of the neck. There was no statistically significant association between gender and headache, between the painful side of the neck and handedness, or between headache and unilateral neck pain.

### 3.3. Comparison of Symptomatic and Non-Symptomatic Side

Table 3 shows the comparative measurements of both sides of the neck of the upper trapezius muscle.

**Figure 7:**
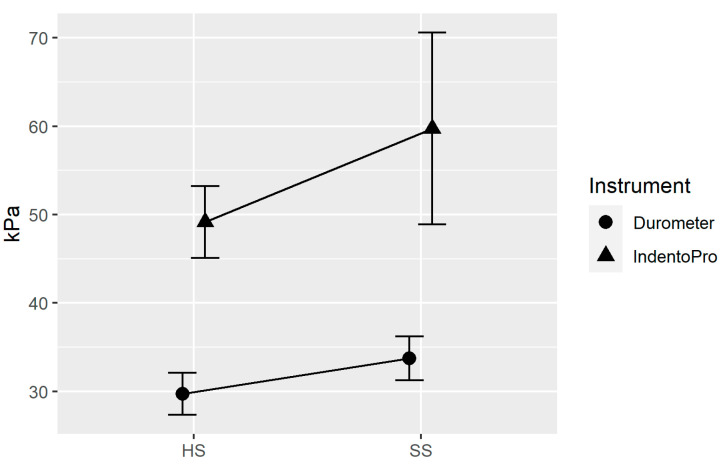
Side-by-side comparison of stiffness of the studied neck sides (error bars show the 95% confidence interval).

The following line plot (Figure 7) shows that both devices used (IndentoPro, durometer) show an increase in stiffness toward the painful side. In other words, there is a statistically significant correlation between stiffness and pain. Looking at both devices, there was a medium Pearson product-moment correlation (r = 0.29, *p* = 0.010) between the two devices, with the durometer presumably measuring mainly superficial tissue layers, while the IndentoPro also reaches deeper myofascial zones [21,28].

There was no significance between the pain symptoms of unilateral neck pain and elasticity. Similarly, there was no correlation between side-dependent pain intensity and PPT. In addition, there was no statistical association between the more painful side of the neck and the opposite side for both lateral flexion and rotation, based on the unilateral function of the upper trapezius.

## 4. Discussion

The key finding of this study is the documentation of increased stiffness of the upper trapezius muscle on the symptomatic side, confirmed by duro- and indentometry. This finding is relevant for future treatment methods, as increased stiffness carries a higher risk of causing hydration dysfunction [29], vasoconstriction, inflammation [30], and further stress injuries [31]. Furthermore, this knowledge can be used to better detect pathological tissue conditions, monitor the effects of therapy and training, and prevent sports injuries [20].

Reasons for the increased stiffness of the selected muscle may include altered tissue morphology of the myofascia and/or possible nerve irritation of the accessorius nerve [32]. The former may be caused by ageing processes [9] or pathogenic changes in the posterior-lateral fascial chain [33], passively transmitted forces from the muscles to the fascial tissue [34], or a fascia-triggered neuromuscular alteration [35] as well as an altered hydration and viscosity state of the fascia [36,37]. An imbalance between the different muscle parts of the trapezius and the prevertebral and spinal extensors is also discussed in the literature as being relevant to increased stiffness. It should be noted that the upper trapezius has been reported as being already anatomically prone to shortening as a posture-dominant muscle [2,3,38]. The significant change in elasticity with age may be explained by age-related processes in the body. From the age of sixty, the number, quality, and strength of muscle fibres decrease, and the collagen content increases, leading to increased stiffness. This effect is more pronounced in women [9,39].

Despite more severe neck pain on one side with a clear localisation, no difference in pain intensity could be measured [40]. This seems to contrast with the subjective perception of the patient. Reduced PPT and increased stiffness at trigger points, including the upper trapezius, have been documented in other studies [41,42]. However, as the present study did not focus on existing trigger points, it is unclear whether the painful side of the neck of the subjects was influenced by an increased expression of such trigger points. A second consideration relates to the assumption of deep nociceptive nerve endings. If these are dampened by the increased measured stiffness of the overlying tissue, this could affect the pressure sensitivity of the upper trapezius muscle, for example, by attenuating it.

The present study has some limitations that warrant future research. The complexity of biomechanical tissue relationships makes it difficult to measure the stiffness of a single muscle, in this case, the upper trapezius. This can be caused not only by pain, but also by surrounding muscles, bony structures, or postural problems of the patient. This ensures that other studies will refute the results of this paper. In particular, the assumption that stiffness should be described objectively, whereas pain is a subjective perception, causes discrepancies in current studies [15]. However, potential measurement errors and bias were largely avoided. Differences are possible in the positioning of the measuring device regarding the angle of pressure and the penetration force exerted when pressing the measuring punch into the myofascial tissue of the trapezius muscle. Due to the different locations, slight variations in the starting position are also realistic, which could also lead to different results. The investigator’s influence is excluded by the blinded procedure, i.e., the investigator had no information about which of the two sides of the neck was reported by the patient to be more painful. The results only refer to one pressure point of the upper trapezius. It would be possible to extend this to other points on the same muscle or other muscle groups that are prone to pain. Another limitation is the age range used. This study included patients up to the age of 75, and it is known that fascia changes with age. There is a degree of inhomogeneity in the subject group, but this reflects clinical reality, as increasing age is associated with increasing pain. This should be considered in future studies and possibly supplemented by a control group. Furthermore, it would also be interesting to find out whether the results can be transferred to other starting positions such as standing or supine [43].

## 5. Conclusions

This study shows unilateral increased stiffness of the upper trapezius muscle on the side of the neck with pain symptoms. However, other myofascial properties such as elasticity and algometry are not significantly altered. Similarly, despite the dominance of pain, there is no homolateral change in mobility of the cervical spine. If confirmed by future additional studies, our current data suggest that a palpatory (or tool-assisted) assessment of myofascial stiffness in the trapezius region may be a recommended feedback tool for evaluating treatments for myofascial neck pain, and most likely more reliable than an associated assessment of pressure tenderness, elastic recoil capacity, or neck mobility.

## Figures and Tables

**Table 1:** Epidemiological data.

Epidemiological Data		Participants (*n* = 40)
Gender (men/women)		7/33
Age (years)	mean ± SD	42.2 ± 14.7
Size (m)	mean ± SD	1.66 ± 0.1
Weight (kg)	mean ± SD	72.9 ± 17.5
BMI (kg/m^2^)	mean ± SD	26.5 ± 5.9
Handedness (right/left)		37/3
Painful side (right/left)		25/15
Pain intensity (VAS)	mean ± SD	5.7 ± 1.5
Headache frequency (0/1/2) ^1^		18/16/6
Headache intensity (0/1/2) ^2^		18/5/15/2

^1^ 0 = none, 1 = irregular, 2 = regular. ^2^ 0 = none, 1 = mild, 2 = moderate, 3 = severe. SD: standard deviation.

**Table 2:** Correlations.

Variable 1	Variable 2	Correlation	*p*-Value *
Durometer	IndentoPro	**0.29 ^1^**	**0.010**
VAS	Headache	0.16 ^2^	0.474
VAS	Headache intensity	0.17 ^2^	0.416
Gender	Headache	0.06 ^2^	1
Gender	Headache intensity	0.08 ^2^	0.700
Handedness	Painful side	0.09 ^2^	0.311
Age	Lateral flexion SS	**−0.47 ^1^**	**0.008**
Age	Lateral flexion HS	−0.31 ^1^	0.203
Age	Rotation SS	−0.30 ^1^	0.233
Age	Rotation HS	−0.25 ^1^	0.491
Age	Elasticity SS	**−0.36** ^1^	**0.048**
Age	Elasticity HS	−0.10 ^1^	1

* Bonferroni corrected. ^1^ Pearson product-moment correlation. ^2^ Somers‘ D. SS: symptomatic side; HS: healthy side. Significant (*p* < 0.05) and at least moderately strong correlations are printed in bold.

**Table 3:** Side-by-side comparison.

Outcome		MW ± SD	95%-CI	*t*-Test (T)	df	*p*-Value *
Algometry (g)	SS	36.41 ± 17.53	30.80–42.02	−0.447	39	0.657
	HS	37.22 ± 17.00	31.78–42.65			
Lateral flexion (°)	SS	37.08 ± 8.15	34.47–39.68	−0.590	39	0.559
	HS	37.73 ± 7.61	35.29–40.15			
Rotation (°)	SS	73.55 ± 12.37	69.59–77.51	0.443	39	0.660
	HS	72.85 ± 11.10	69.30–76.40			
Durometer (kPa)	SS	33.76 ± 7.78	31.27–36.25	**4.995**	**39**	**<0.001**
	HS	29.75 ± 7.45	27.37–32.13			
IndentoPro (kPa)	SS	59.73 ± 33.93	48.88–70.59	**2.352**	**39**	**0.024**
	HS	49.18 ± 12.69	45.12–53.23			
Elasticity (N/mm)	SS	0.101 ± 1.09	−0.246–0.448	−0.822	39	0.416
	HS	-0.006 ± 0.29	−0.098–0.087			

* Bonferroni corrected. SS: symptomatic side; HS: healthy side. Significant results (*p* < 0.05) are printed in bold.

## Data Availability

The datasets used and/or analysed in the current study or any query regarding the research process are available from the corresponding author.
